# Heterogenized Imidazolium-Based Ionic Liquids in Pebax^®^Rnew. Thermal, Gas Transport and Antimicrobial Properties

**DOI:** 10.3390/polym12061419

**Published:** 2020-06-25

**Authors:** Gabriele Clarizia, Paola Bernardo, Sabrina C. Carroccio, Martina Ussia, Cristina Restuccia, Lucia Parafati, Anna Calarco, Daniela Zampino

**Affiliations:** 1 Institute on Membrane Technology, ITM-CNR (c/o University of Calabria), Via P. Bucci 17/C, 87036 Rende (CS), Italy; g.clarizia@itm.cnr.it; 2 Institute of Polymers, Composites and Biomaterials, IPCB-CNR, via P. Gaifami 18, 95126 Catania, Italy; sabrinacarola.carroccio@cnr.it; 3 Institute for Microelectronics and Microsystems, IMM-CNR (c/o University of Catania), Via Santa Sofia 64, 95123 Catania, Italy; martina.ussia@gmail.com; 4 Department of Agriculture, Food and Environment (Di3A), University of Catania, Via Santa Sofia 100, 95123 Catania, Italy; crestu@unict.it (C.R.); lucia.parafati@hotmail.it (L.P.); 5 Research Institute on Terrestrial Ecosystems, IRET-CNR, Via P. Castellino 111, 80131 Napoli, Italy; anna.calarco@cnr.it

**Keywords:** imidazolium-based ionic liquids, polymeric films, antimicrobial activity, gas transport

## Abstract

Imidazolium-based ionic liquids (ILs) have interesting antimicrobial activity and their inclusion in a flexible film is ideal to take advantage of their properties in practical applications. Poly(ether-block-amide) (Pebax^®^Rnew) films were prepared by solution casting, loading two synthetized ILs (1-hexadecyl-3-methylimidazolium dimethyl-5-sulfoisophthalate [Hdmim][DMSIP], IL1 and 1-octyloximethyl-3-methylimidazolium hexafluorophosphate [OOMmim][PF_6_], IL2) up to 5 wt.%. The ILs were characterized by ^1^H NMR and MALDI-TOF spectroscopy. The films were investigated for miscibility, morphology, wettability, spectral properties and gas transport. The films display a good thermal stability (>200 °C). Differential scanning calorimetry (DSC) proves phase separation in the blends, that is consistent with FTIR analysis and with the island-like surface morphology observed in the micrographs. Gas permeability tests revealed that the IL-loaded films are dense and poreless, keeping the selectivity of the polymer matrix with a somewhat lessened permeability owing to the impermeable ILs crystals. The film antimicrobial activity, evaluated against Gram-negative and Gram-positive bacterial strains, was correlated to the structure of the incorporated ILs. The smaller IL2 salt did not modify the hydrophobic nature of the neat polymer and was readily released from the films. Instead, IL1, having a longer alkyl chain in the cation, provided a promising antimicrobial activity with a good combination of hydrophilicity, permeability and thermal stability.

## 1. Introduction

Bacterial multiresistance is one of the main problems that humanity has to face. The emergence of antibiotic-resistant foodborne bacteria in recent times calls for concerted efforts and alternative strategies since foodborne diseases acquired from contaminated cooked, raw, processed or unprocessed food are causes of mortality and morbidity globally [1]. Antimicrobial properties are required in food packaging and biomedical devices since microbial contamination can cause serious problems for public health and safety [2]. Ionic liquids (ILs) are salts displaying a melting point of up to 100 °C and a low vapor pressure combined with a remarkable synthetic flexibility to design their physicochemical properties [3]. Different studies demonstrated antimicrobial activity of ILs comprising cations based on the imidazole heterocycle (antibacterial, antifungal, antitumor, antioxidant and antifibrous agents) [4,5,6]. The insertion of cations into the lipid bilayer cell membranes of microorganisms [7] causes their structural damage [8]. Therefore, the toxicity of the ILs can be tuned and exploited for beneficial uses for contamination and infection control in the environment. In particular, N-Alkylimidazolium-based ILs are recognized as promising antimicrobial agents [9], particularly those with long alkyl chains [10,11]. Raucci et al. reported that multifunctional calcium phosphate (CaP)-IL materials, based on the loading of 1-alkyl-3-alkylimidazolium chloride ionic liquids (ILs) (CnMImCl (*n* = 4, 10, 16) and (C_10_)_2_MImCl) during the in situ sol−gel synthesis of calcium phosphates (CaP), displayed an antimicrobial activity that increased with an increasing N-alkyl chain length: C_4_MImCl < C_10_MImCl < C_16_MImCl [6]. N-hexadecyl was identified as an optimum N-alkyl chain length, demonstrating a broad-spectrum toxic effect; this behavior was related to an ideal amphiphilic nature of the 1-n-hexadecyl-3-methylimidazolium cation [12]. In addition, ILs can help in dispersing nanofillers within polymeric matrices [13].

Heterogeneous assemblies can exploit the properties of ionic liquids as biocidal agents [14]. Several studies focused on “liquid supported membranes” in which porous supports of polymeric materials are as filled with the ILs and adopted as separating membranes [15]. The preparation of these materials as polymeric gels entrapping the IL molecules would overcome leaching issues typically reported for “liquid supported membranes” [16]. Simple blending of ILs and selected polymers is a low-cost and less time-consuming procedure with respect to the polymer grafting. The addition of ILs within a polymeric matrix via covalent bonding assures a sufficient stability. However, blending usually results in better mechanical behavior than polymerized ILs [17].

Pebax^®^ are thermoplastic block copolymers applied for medical uses (e.g., short-term implantation in humans and virus-proof surgical sheeting) [18]. These macromolecules include linear chains of hard polyamide (PA) blocks covalently linked to soft polyether (PE) blocks via ester groups. Different types of the PE and PA segments and their relative ratio in the copolymers result in diverse properties. Pebax^®^Rnew comprises Polyamide 11 (PA11) as a hard block that is bio-based since it is obtained from renewable raw materials (castor seeds). The physical and mechanical properties of the copolymer depend on the hydrogen bonding between amide (–CONH–) groups in adjacent chains. The polar PA11 blocks can also interact via hydrogen bonds with smaller polar molecules such as the ILs. Being rubbery, Pebax^®^Rnew would result in flexible films, without requiring the use of harmful plasticizers [19], as in the case of polyvinyl chloride (PVC), which is widely used to manufacture equipment for medical devices [20]. 

The present study focuses on blends of the commercial polymer Pebax^®^Rnew and two ILs (1-hexadecyl-3-methylimidazolium dimethyl-5-sulfoisophthalate [Hdmim][DMSIP], IL1 and 1-octyloximethyl-3-methylimidazolium hexafluorophosphate [OOMmim][PF_6_], IL2). They present the imidazolium group in the cation and differ for the alkyl chain and the anion in order to investigate the effect of a different structure on their antimicrobial activity. In particular, the anions were selected in order to have different hydrophobicity/hydrophilicity properties. Dense films were prepared by solution casting, immobilizing the IL salts into the polymer matrix, using ethanol as common solvent. The novel Pebax/ILs blend system was investigated for their compatibility and the self–supported membranes were tested in gas permeation to investigate the membrane microstructure by using the gases as molecular probes. Their antimicrobial properties were examined against Gram-negative (*Escherichia coli*, *Pseudomonas fluorescens*, and *Salmonella enterica*) and Gram-positive (*Listeria monocytogenes* and *Bacillus subtilis*) bacteria as well as their toxicity for potential applications in antimicrobial surface coating. 

## 2. Experimental

### 2.1. Materials

Pebax^®^Rnew 25R53, a block copolymer consisting of flexible poly(tetramethylene oxide) (PTMO) and rigid PA11, was received in pellets from Arkema, Italy. Ethanol (absolute, VWR) was used as solvent for the preparation of the films.

1-methylimidazole, 1,3-dimethyl 5-sulfoisophthalate sodium salt, 1-bromohexadecane, chloromethyl octyl ether, sodium hexafluorophosphate, dichloromethane (DCM), ethyl acetate, tetrahydrofuran (THF), hexane, dimethyl sulfoxide-d6 (DMSO-d6), trans-2-[3-(4-tert-butylphenyl)-2-methyl-2-propenylidene]malononitrile, 1-decyl-3-methylimidazolium chloride (Dmim Cl), sodium p-toluenesulfonate and ammonium acetate were supplied by Sigma-Aldrich and used as received.

Gases for permeation tests are: O_2_, N_2_, CH_4_ and CO_2_ (purity of 99.99+%) from SAPIO, Italy. 

Fetal bovine serum (FBS), Roswell Park Memorial Institute (RPMI) 1640 medium, Dulbecco’s modified Eagle’s medium (DMEM), sodium pyruvate, l-glutamine, penicillin and streptomycin were purchased from Hyclone (Milan, Italy).

### 2.2. Methods

#### 2.2.1. ILs Synthesis

**Figure 1:**
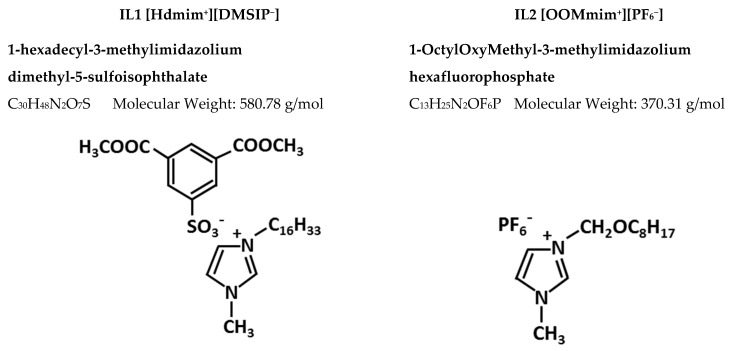
The chemical structures of the synthetized ILs.

The chemical structure of the ILs is reported in Figure 1; their synthesis is described below. Both ILs were synthetized by alkylation of 1-methylimidazolium, yielding the formation of ILs halides, and then by ionic exchange between the halides and sodium salts the desired ILs were obtained.

##### **1-hexadecyl-3-methylimidazolium dimethyl-5-sulfoisophthalate ([Hdmim][DMSIP], IL1) synthesis** (according to Colonna *et al*., slightly modified [10]).

The first step of the synthesis was performed by reacting 1-methylimidazole (1.56 mL, 20 mmol), dissolved in ethyl acetate (4 mL), with 1-bromohexadecane (6.4 mL, 20 mmol) under nitrogen atmosphere, for 24 h at 65 °C, yielding the formation of hexadecyl-3-methylimidazolium bromide. The obtained compound was filtered, washed with ethyl acetate to take out unreacted initial products, and dried under vacuum at 40 °C for 24 h (yield 95 %). 

^1^H NMR (200 MHz, DMSO-d6, δ ppm): 0.84 (t, 3H, CH_3_-C15), 1.23 (m, 26H, CH_2_), 1.77 (m, 2H, CH_2_-CH_2_-N), 3.85 (s, 3H, CH_3_-N), 4.15 (t, 2H, CH_2_-N), 7.72 (s, 1H, CH in imidazolium ring), 7.78 (s, 1H, CH in imidazolium ring), 9.15 (s, 1H, N-CH-N in imidazolium ring). 

The second step of the synthesis involved a metathesis reaction between a dichloromethane (DCM) solution (40 mL) of 1-hexadecyl-3-methylimidazolium bromide (20 mmol, 7.77 g) and a water solution (130 mL) of 1,3-dimethyl 5-sulfoisophthalate sodium salt (20.9 mmol, 6.03 g) by vigorously shaking at 25 °C (30 min) until separation of mixture in two clear phases. The organic layer was taken, dried over magnesium sulfate and the residual solvent was removed by a rotavapor. The obtained compound was washed with ethyl acetate and dried under vacuum for 24 h at 40 °C (yield 92 %). The complete exchange of the bromide counter-ion was verified by a silver nitrate test. 

^1^H NMR (400 MHz, DMSO-d6, δ ppm): Signals of imidazolium ring and alkyl chain: 0.84 (t, 3H, CH_3_-C_15_ chain), 1.22 (m, 26 H, CH_2_), 1.75 (m, 2H, CH_2_-CH_2_-N), 3.83 (s, 3H, CH_3_-N), 4.14 (t, 2H, CH_2_-N), 7.69 (s, 1H, CH in imidazolium ring), 7.75 (s, 1H, CH in imidazolium ring), 9.10 (s, 1H, N-CH-N in imidazolium ring). 

Signals of benzene ring: 3.90 (s, 6H, CH_3_-O), 8.37 (d, 2H, CH, ortho-position with respect to SO_3_- substituents), 8.42 (d, 1H, CH, para-position with respect to SO_3_- substituents).

##### **1-octyloxymethyl-3-methylimidazolium hexafluorophosphate ([OOMim][PF_6_], IL2) synthesis** (according to Pernak et al. [21]).

Chloromethyl octyl ether (3.86 mL, 0.02 mol, 3.57 g) and 1-methylimidazole (1.585 mL, 0.02 mol, 1.6 g) were stirred together under nitrogen atmosphere, at room temperature, for 30 min. The obtained 3-octyloximethyl-1-methyl imidazole chloride was purified by washings with hot hexane (at 50 °C). In the second step of reaction, two water solutions (10 mL each) of sodium hexafluorophosphate (0.02 mol, 3.36 g) and 1-octyloxymethyl 3-methyl imidazole chloride (0.02 mol) were reacted under stirring on an oil bath at 50 °C for 2 h. The organic fraction was separated by centrifugation at 3000 rpm for 5 min and dried under vacuum at 40 °C for 48 h (yield 95 %). 

^1^H NMR (400 MHz, DMSO-d6, δ, ppm): 0.85 (t, 3H, CH_3_–C_7_ chain), 1.23 (m, 10 H, CH_2_), 1.48 (m, 2H, CH_2_-CH_2_-O), 3.47 (s, 3H, CH_2_-O), 3.88 (t, 2H, CH_3_-N), 5.54 (s, 2H, O-CH_2_-N), 7.76 (s, 1H, CH in imidazolium ring), 7.85 (s, 1H, CH in imidazolium ring), 9.28 (s, 1H, N-CH-N in imidazolium ring).

#### 2.2.2. Membrane Preparation

Pebax^®^ was dissolved in ethanol at 2 wt.% concentration and then mixed in different amounts with the ILs. The polymer dissolution required the heating under reflux conditions for ca. 2 h.

The ILs were both solid at room temperature. They were loaded at a concentration of 1 and 5 wt.%, by heating the polymer solution at ca. 50 °C.

The membranes were prepared as dense flat sheets by solution casting the polymer/IL mixture within a stainless-steel ring placed onto a Teflon support. The casting ring was left at room conditions overnight for a slow solvent evaporation. Isotropic films based on the neat polymer were prepared as well and used as references.

### 2.3. Characterization

#### 2.3.1. NMR Spectroscopy

^1^H NMR spectra were recorded at 20 °C on a Bruker Advance 400 spectrometer using the TOPSPIN 2.1 acquisition software. Samples (10 mg/mL) were dissolved in DMSO-d6. 

#### 2.3.2. Matrix Assisted Laser Desorption Time of Flight Mass Spectrometry (MALDI TOF MS) Analysis

MALDI mass spectra were acquired in reflector mode by a 4800 MALDI TOF/TOF™ Analyzer (Applied Biosystem, Framingham, MA, USA), equipped with a Nd:YAG laser (wavelength of 355 nm) and working in positive-ion mode. The laser had a wavelength of <500 ps pulse and 200 Hz repetition rate. Mass resolution of MALDI spectra was about 10,000 (full width at half maximum, FWHM), and mass accuracy was 1–10 ppm for masses in the range m/z 200–1000 Da. Trans-2-[3-(4-tert-Butylphenyl)-2-methyl-2-propenyldene] malononitrile (0.1 mmol in THF) was used as a matrix. Appropriate volumes from solutions of ionic liquids were dissolved in THF (10 mg/mL concentration) and were mixed with the matrix to obtain 1:1, 1:2 and 2:1 ratios (sample/matrix *v*/*v*). 1 µL of each sample/matrix mixture was spotted onto the MALDI sample holder and dried at 25 °C to allow matrix crystallization. The structural identification of MALDI peaks was made based on empirical formulas [22]. 

#### 2.3.3. Calorimetric Analyses

Calorimetric measurements were carried out using a differential scanning calorimetry (DSC, TA Instruments Q100), equipped with a sub-ambient accessory. Temperature and heat flow were calibrated with high purity standards (indium and cyclohexane). Samples of ca. 4–5 mg were wrapped in aluminium pans and subjected to heating/cooling/heating cycles. An empty aluminium pan was used as a reference. All the heating and cooling runs were performed at a rate of 10 °C/min in the temperature range of −90 to 250 °C under nitrogen atmosphere. 

#### 2.3.4. Thermogravimetric Analyses (TGA)

The thermogravimetric analyses were performed in a thermogravimetric apparatus (TGA, TA Instruments Q500) under nitrogen at a purge flow of 60 mL/min. Samples (4–5 mg) were heated from 40 °C to 800 °C, at a rate of 10 °C/min. 

#### 2.3.5. Fourier Transform Infrared (FTIR)

Functional groups of the prepared films were investigated by attenuated total reflection FTIR analysis. The spectra were recorded in a FTIR Spectrum One (Perkin Elmer), in the region from 4000 to 650 cm^−1^, with a resolution of 4 cm^−1^ (10 scans).

#### 2.3.6. Scanning Electron Microscopy (SEM)

SEM analysis was carried out using a Gemini Field Emission SEM (FE-SEM) Carl Zeiss SUPRA 25. All samples were previously covered with 5 nm thickness of gold using the sputtering method.

#### 2.3.7. Contact Angle (CA)

The wettability of samples was analyzed at 25 °C by using a DATAPHYSICS-OCA 15 PRO apparatus. The contact angles values were measured in three different areas of the same sample. Specifically, a deionized water drop volume of 4 µL was formed on polymeric surfaces whose volume was controlled by using the software of the optical tensiometer. When the drop appeared stable (~10 s), three different measurements were done for each volume and each sample. Contact angle results were reported as average values with a standard deviation of ±2°.

#### 2.3.8. Gas Permeation Tests

Gas permeation tests on flat dense membranes were carried out at with single gases (H_2_, He, N_2_, O_2_, CH_4_ and CO_2_) in a fixed volume/pressure increase instrument (Elektro & Elektronik Service Reuter, Germany) at 25 °C [23]. Before each experiment, the membrane samples were carefully evacuated using a turbo molecular pump to remove previously dissolved species. The membrane was exposed to the feed gas at a pressure of 1 bar, following the pressure increase over time in a calibrated volume at the permeate side. The gas permeability *P* was obtained from the slope of the pressure curve at steady state condition. The extrapolation of the linear portion of the pressure on the abscissa provides the gas time lag (*θ*) and thus the diffusion coefficient, *D*, of each gas through the membrane [24]:

(1) $$D=\frac{l^{2}}{6\theta }$$

where *l* is the membrane thickness.

The ideal selectivity was calculated as the ratio of the individual permeability values for two gases and can be decoupled into solubility selectivity and diffusivity selectivity:

*α*_A/B_ = *P*_A_/*P*_B_ (2)

Circular samples having an effective area of 11.3 or 2.14 cm^2^ were tested. The membrane thickness was obtained as the average of multiple point measurements made with a digital micrometer (IP65, Mitutoyo).

#### 2.3.9. Antimicrobial Activity

The potential antimicrobial activity of the above mentioned films was evaluated using the disk diffusion method, which basically consists of measuring the growth inhibition halo produced by the film attached to a plate of nutritive medium previously inoculated with the target microbial strain. 

Overnight bacterial cell suspensions (100 µL at a concentration of 10^6^ CFU/mL) of *Escherichia coli*, *Pseudomonas fluorescens*, *Salmonella enterica*, *Listeria monocytogenes* and *Bacillus subtilis* strains, were individually spread on the surface of Petri plates containing Nutrient Agar (NA, Oxoid, Basingstoke, UK) medium. The Pebax^®^Rnew/ILs films containing amounts of ILs at 1 and 5 wt.% were sterilely laid on the plate surface and allowed to dry for five minutes.

The control plates were made using Pebax^®^Rnew films containing 0 wt.% of ILs. All plates were then incubated at 30 ± 2 °C for 24–48 h. The inhibitory effect of the film against target strains was assessed measuring the size (mm) of the inhibition zone (no bacterial growth) around the film. Each test was performed in triplicate.

#### 2.3.10. IL Release

The ILs release was determined by immersing round samples (4 cm^2^ surface) of neat polymer membranes and films loaded with 5% of ILs in glass tubes containing sterile water. Samples (at least three replicate specimens) were incubated at 37 °C and removed after 30 min, 1, 5, 8 and 24 h. The water media from sampling were used for the determination of the ILs released during the fixed time period. 

The concentration of ILs in the release medium was determined via direct infusion by using an UHPLC system, coupled to an Orbitrap MS single-stage (Exactive™, Thermo Fisher Scientific, Bremen, Germany) and operating with an electrospray interface (HESI-II, Thermo Fisher Scientific). Samples (1 μL) directly introduced into the mass spectrometer were subjected to an isocratic elution at a flow rate of 0.1 mL/min using a mobile phase consisting in ACN/H_2_O 50/50 *v*/*v*. 

The concentration of ILs cations and anions was determined through the construction of their related calibration curves, using the internal standard method for Hdmim and OOMmim cations and DMSIP anion, and the external standard method to quantify PF_6_ anions. 1 ppm of each of the internal standards (IS) was employed for the construction of calibration curves, using 1-decyl−3-methylimidazolium chloride for the determination of HDmim and OOMmim cations and p-toluene sulfonate for the DMSIP anion.

#### 2.3.11. Cell Culture and Cytotoxicity Assay

The human adenocarcinoma cells (Caco-2) were obtained from the American Type Culture Collection (ATCC). Cells were maintained in DMEM supplemented with 10% FBS, 1% l-glutamine, 50 U/mL penicillin and 50 mg/mL streptomycin at 37 °C in a humidified atmosphere containing 5% CO_2_. Cells were tested for contamination, including mycoplasma, and used within 2 to 4 months.

To assess ILs cytocompatibility, a Cell Counting Kit-8 (CCK-8) assay was carried out as reported by Conte et al. [25]. 24 h before treatment, cells were seeded in a 96-well plate in 100 μL culture medium at a density of 4 × 10^3^ cells/well. Then, cells were treated with release medium of the films serially diluted by a factor of 10 and incubated at 37 °C for 3, 6, 12, 24 and 48 h. The films characterized were: neat Pebax and those with 5% of IL1 or IL2. Then, 10 μL of CCK-8 solution were added to each well, and the plate was incubated under cell culture conditions for 1–4 h. The optical density of formazan salt at 450 nm was measured using a Cytation 3 Cell Imaging Multi-Mode microplate reader (Biotek, Milan, Italy). ILs cytocompatibility was expressed as a percentage relative to the control and calculated by the equation:

Cytocompatibility (%) = (OD_sample/OD_control) × 100(3)

where OD_sample is the optical density of cells treated with PHB-PEI NPs and OD_control is the optical density of untreated cells.

## 3. Results

### 3.1. ILs Synthesis and Characterization and Membrane Preparation

The ILs were synthetized by a two steps technique, involving the alkylation of 1-methylimidazole and a metathesis reaction between DCM/water solutions of the bromide/chloride obtained and the inorganic salts selected, with yields of 92% and 95%, respectively. The imidazolium salts obtained were low-melting solids at room temperature. 

^1^H NMR spectra allowed us to determine the successful functionalization of the imidazole ring. In particular, the ^1^H-NMR spectrum of IL1 (Figure S1, Supporting Information) shows the characteristic peaks of the synthetized salt, also confirming that the metathesis reaction occurred. Moreover, the analysis of the MALDI-TOF spectrum (Table 1; Figure S2), showing the peak of cationic species at 307.34 and an adduct (at 887.77 m/z) made of the cationic species with the [Hdmim][DMSIP] IL, corroborated the effective outcome of the synthesis. In the case of the IL2, ^1^H-NMR spectrum proved that the alkylation and quaternization of imidazolium ring happened through the signals relative to OOMmim cation formation (Figure S3). The metathesis reaction between OOMmim Cl and sodium hexafluorophosphate was confirmed by MALDI-TOF spectrum (Table 1, Figure S4) through the signal at 225.19 m/z, relative to OOMmim cationic species, and the mass peak at 595.36 m/z, corresponding to an adduct constituted by the sum of the cationic species with the [OOMmim][PF_6_] IL. 

Macroscopically homogeneous and freestanding films were obtained with a thickness in the range 80–120 micron. The first macroscopic feature of the blends is their easy handling. This character is particularly interesting for applications such as flexible packaging. 

### 3.2. Thermal Analysis

To evaluate the thermal stability of ILs, neat Pebax^®^Rnew and Pebax^®^Rnew/ILs blends, DSC and TGA measurements were carried out. 

#### 3.2.1. DSC Measurements

DSC curves (Figs. SI 5 and SI 6) showed melting (T_m_) and crystallization (T_c_) temperatures of 49 °C and −4 °C for IL1 and 52 °C and 7 °C for IL2, respectively. The comparison of the ILs heating scans showed only a slight difference of 2–3 °C of T_m_ from the first to second heating cycles. In the case of IL1, however, we observed a polymorphic behavior on the second heating scan, characterized by a cold crystallization peak (Tcc), similar to that showed by polymer materials. Moreover, endothermic transitions of overlapped peaks, probably associated with melting of different crystals or solid–solid transition followed by recrystallization, which immediately melted, were observed, and were in agreement with thermal behavior of other ILs [26].

**Figure 2:**
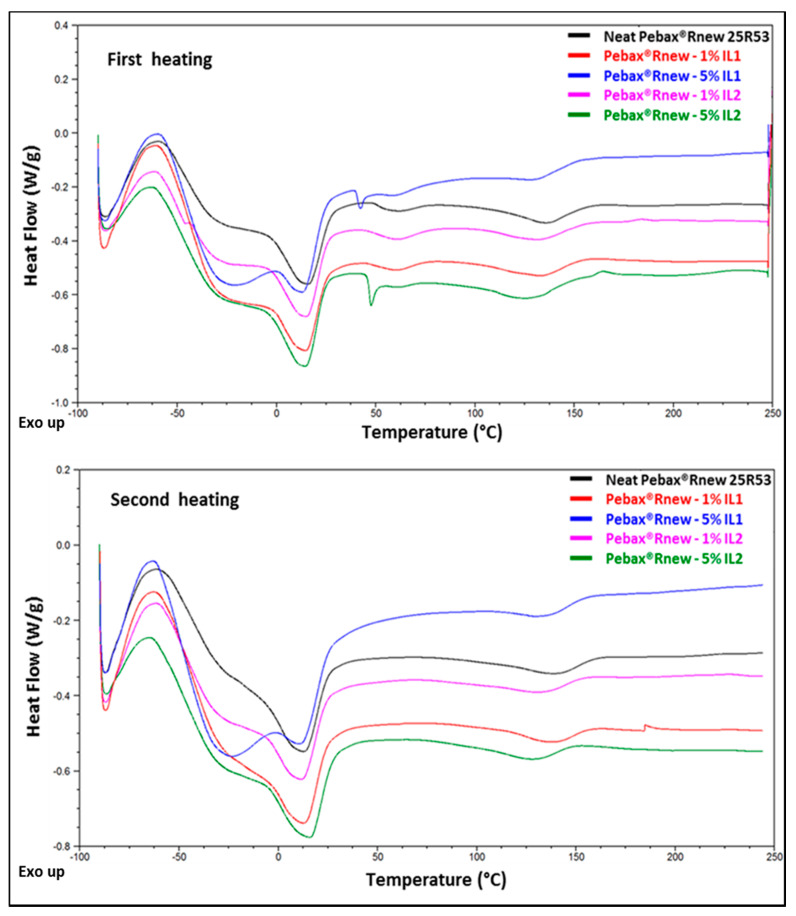
DSC thermograms (first heating and second heating runs) of Pebax^®^Rnew and Pebax^®^Rnew/ILs blends.

DSC analysis of neat Pebax^®^Rnew and Rnew/ILs blends (Figure 2) showed two main melting peaks relating to both PTMO and PA11 blocks, proving the microphase separated structure of the block copolymer. In the first heating cycle of neat Rnew, the melting temperature (*T*m) of the polyether block was 17 °C, whereas that of PA-11 was 137 °C. The blends containing 5 wt.% of ILs showed melting peaks at 42 °C and 48 °C, relative to *T*m of IL1 and IL2, respectively. A broad peak, likely due to a kinetically less favorable crystal phase caused by the slow solvent evaporation during membrane preparation, was observed at ca. 60 °C in all samples. During the second heating run, this peak at ca. 60 °C disappeared together with the melting peaks of ILs. Therefore, a crystalline phase is present in the films formed from the solution state starting from solid ILs, while a different result is achieved from the molten state. A slight shift to a lower temperature (13 °C) was noted for *T*m of the PTMO block, while a shift to a high temperature (142 °C) was observed for PA block. An additional endothermic transition was detected at ca. −25 °C in both heating runs, particularly in the blend with 5% of IL1.

The *T*m and enthalpy of fusion (∆*H*m) of PTMO and PA blocks in the second heating run of Pebax^®^Rnew and Pebax^®^Rnew/ILs blends are reported in Table 1. Both ILs induced a reduced crystallinity for the predominant PTMO phase (reflected in a decrease of Δ*H*m that is more than the decreasing polymer concentration in the Pebax/IL blend). However, the PTMO has a melting point below room temperature (23–25 °C) and is thus amorphous at higher temperatures. In general, ILs addition into Pebax^®^Rnew matrix did not influence the position of melting and crystallization peaks; only slight shifts (1–5 °C) were detected, suggesting no interaction and consequently phase separation between the polymer and the ILs [16]. 

#### 3.2.2. TGA

**Figure 3:**
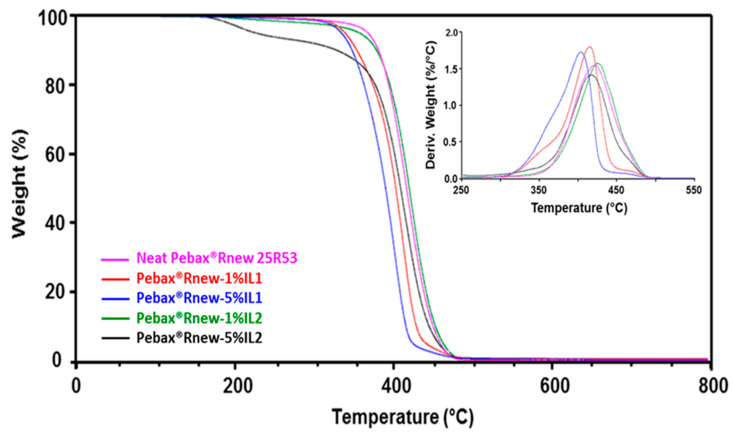
Thermogravimetric and DTG (inset) curves of Pebax^®^Rnew and Pebax^®^Rnew/ILs blends.

Figure 3 shows an overlay of thermogravimetric and DTG curves (inset) of neat Pebax^®^Rnew and its blends. The mass losses (TG) obtained from thermogravimetric analysis of neat Pebax^®^Rnew and its blends are presented in Table 2. 

Both ILs displayed a single step degradation, with maximum degradation temperatures at 411 °C and at 271 °C for IL1 and IL2, respectively. TGA data relative to onset temperature, i.e., degradation start temperature, showed that IL1 is more stable (ca. 340 °C) than IL2 (ca. 240 °C), most likely due to the hygroscopic behavior of IL2. Decomposition temperatures of ILs mainly depend on the anion coordinating nature rather than on the alkyl chain length of the cation. Indeed, the thermal stability of poor coordinating anions such as PF_6_ is higher than that of high coordinating halide anions (Cl, Br) [27,28]. The hygroscopic behavior of IL2 affected its thermal stability though rigorous drying protocols were carried out. 

Neat Pebax^®^Rnew showed one-step decomposition concerning the random chain scission mechanism of the main polymer chain. The blends also showed a single-step degradation, suggesting that the presence of ILs does not influence the thermal degradation pattern of the polymer. No significant differences between neat Pebax^®^Rnew and Pebax^®^Rnew/ILs blends were observed at below 200 °C, whereas at temperatures higher than 200 °C, all the blends showed a decrease of the onset temperature. In particular, the addition of IL1 to the polymer matrix led to a ca. 30 °C decrease of the onset temperature, while this difference is of 12 °C for Rnew/1% IL2 and unexpectedly more than 100 °C for the blend containing 5% of IL2. This low value is due to the presence of residual solvent and absorbed water trapped into the polymer blend, even after two vacuum drying cycles at 80 °C. The persistence of water and solvent into matrix of this blend, confirmed by DTG, showing a peak at 160 °C, is probably caused by the IL2 high hygroscopic behavior, enhanced by the slow solvent evaporation at room temperature during membrane preparation. 

The temperature of the maximum degradation rate (*T*d) was not affected by the addition of ILs into Pebax^®^Rnew matrix, displaying values higher than 400 °C for all the blends. These results are in agreement with literature data. In a study on imidazolium PBT ionomers preparation, Colonna et al. [10] found that thermal stability of ionomers was similar to pristine PBT, only showing a slight decrease up to a maximum of 8 °C with ionic content. On the contrary, Rogalsky and co-authors [29] found a lowering of thermal decomposition with the introduction of ILs into the polycarbonate (PC) matrix. They observed that pure PC showed a single step degradation and no weight loss up to 400 °C, while PC/IL compounds displayed a two-step degradation process with lowered thermal degradation points due to products of IL initial thermal degradation. In any case, the prepared films can be subjected to sterilization (typically at 150 °C). 

No residue was observed at 800 °C because all the samples showed end of degradation within 550–600 °C (Figure 3).

#### 3.2.3. FTIR

FTIR spectra are gathered in Figure S7. Molecular interaction between the polymer and the ILs was not observed. The distinct peak at ca. 1100 cm^−1^ was assigned to the stretching vibration of C-O-C group within the PTMO block in the neat Pebax. The polyamide segment in Pebax showed relatively sharp peaks at around 3309, 1637 and 1735 cm^−1^ attributed to the -N-H-, H-N-C=O and O-C=O groups, respectively. In the IL2-loaded film, the clear band appearing at 844 cm^−1^ was attributed to the stretching vibration of the PF_6_^−^ anion. 

#### 3.2.4. SEM

**Figure 4:**
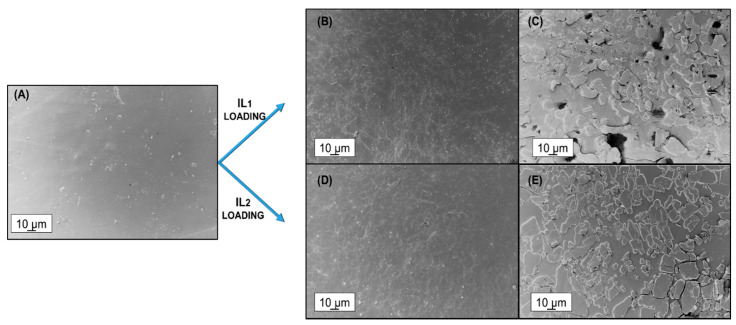
SEM images taken on the surface of Pebax^®^Rnew and the blends with 5 wt.% ILs. (**A**) Neat Pebax^®^Rnew; (**B**) Pebax^®^Rnew/1% IL1; (**C**) Pebax^®^Rnew/5% IL1; (**D**) Pebax^®^Rnew/1% IL2; and (**E**) Pebax^®^Rnew/5%IL2.

SEM micrographs were taken on the surface of the membranes and shown in Figure 4. Pebax^®^Rnew displayed a smooth surface (Figure 4A). As it is possible to notice from the micrographs, the 1% of addition for both did not significantly change the morphology of matrix sample, presenting merely a slightly increment of the roughness. Conversely, both film blends at 5% of ILs (IL1, Figure 4C and IL2, Figure 4D) showed discontinuous and more roughly morphologies, consisting of island-like structures as also observed by Xi et al. [30]. At this concentration, a decrease in compatibility with the polymer matrix was experienced determining a surface segregation with formation of microstructures with sizes ranging from 5 to 20 µm. Data collected by SEM analysis supported the DSC measurements previously discussed.

#### 3.2.5. Contact Angle

**Figure 5:**
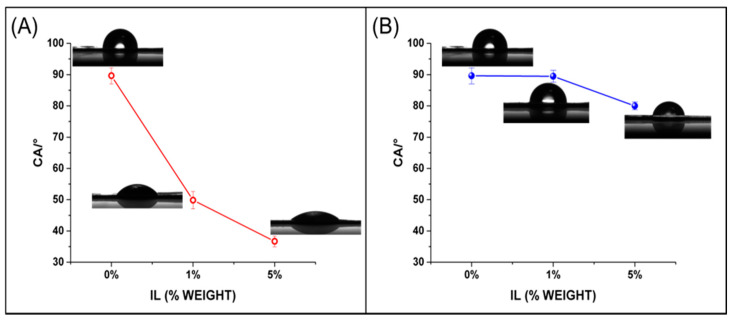
Water contact angles for Pebax^®^Rnew and Pebax^®^Rnew/ILs blends. (**A**) IL1 blends and (**B**) IL2 blends.

Water contact angles of unloaded Pebax^®^Rnew and Pebax^®^Rnew/ILs blend films were evaluated to investigate their wettability (Figure 5). 

The films of neat Rnew polymer resulted hydrophobic, displaying a water contact angle of 89.6° (>65° [31]). By adding 1% and 5 wt.% of IL1, Pebax^®^Rnew blend films became more hydrophilic with a contact angle decreasing significantly from 89.6° to 50°, then down to 38° (Figure 5A). Instead, Rnew/IL2 films had almost constant contact angle values upon the introduction of 1 wt.% of IL with a slight decrease at 5 wt.% of IL2 (Figure 5B). These data indicated the possibility to tailor the wettability, depending on the IL loaded in the Pebax^®^Rnew matrix. In our case, the contribution of ILs nature is more significant than surface roughness, as can be deduced by comparing the films at low IL concentration (1 wt.%). This phenomenon can be associated to both cations and anions structures in ILs. Huddleston et al. reported [28] that hydrophobicity of cations increases with the increase of the CH_2_ groups in the alkyl chain linked to the nitrogen of the imidazolium ring, while in the case of anions, it depends on the presence of moieties able to establish hydrogen bonds (with donating or accepting ability). The structure of the IL1 head group, bearing a C_16_ alkyl chain, makes the cation more hydrophobic; nevertheless, this effect is counterbalanced by the coordinating ability of its anion, higher than that of PF_6_. Conversely, IL2 having a cation with a shorter alkyl chain and a more hydrophobic anion only slight influences the wettability of Rnew. The anion chemistry has a large influence on the properties of IL. Indeed, in our case, as PF_6_^−^ (in IL2) is more hydrophobic [28] and DIMSIP^−^ (in IL1) is more hydrophilic, the anion type determined the membrane wettability. However, we cannot exclude the contribution of long alkyl chain present in the IL1 to improve the miscibility with the Rnew because it is constituted of long alkylic spacers among the amide groups of PA11 block.

In general, approaches to reduce protein adsorption on a film include surface modification in order to promote a hydrophilic behavior [32]. Therefore, the gain in hydrophilicity obtained upon the inclusion of IL1 in the films is highly desirable to obtain anti-fouling devices.

#### 3.2.6. Gas Permeation

Permanent gases can be considered as molecular probes providing information on the membrane microstructure. The gas permeation tests provided the following transport parameters: permeability, ideal selectivity as reported in Table 3. These data confirm the dense, poreless nature for all films. 

The neat Pebax is quite permeable, with permeability values close to those reported for Pebax^®^2533 [23]. These results depend on the large amount of soft PE blocks that, owing to their high chain mobility, are gas permeable, while the hard PA segments provide mechanical stability.

The addition of ILs to Pebax^®^ results in films with a reduced gas permeability with respect to the neat polymer. The data suggest a microstructure in the blends without the formation of large cavities that would function as bypasses for the tested gases. These defects, resulting in larger permeability and in a selectivity loss, would deteriorate the barrier performance of the film. The permeability reduction was more pronounced using the IL1 that presents larger molecular weight and molar volume than IL2. Indeed, both ILs are solid at room temperature, therefore, they act as impermeable fillers [33]. According to the DSC analysis, the preparation conditions for the membranes as well as the testing temperature are below the melting of the ILs that remains as crystals in the films. At same time, operating above the PTMO melting, it is not possible to detect the plasticizing effect of the ILs seen in the thermal analysis.

In both blend membranes, CO_2_ was the most permeable species among the tested gases, thus keeping the behavior of the Pebax matrix. Studies on sorption and permeation properties of Pebax copolymers evidenced strong interactions between the polar CO_2_ gas and the Polyether blocks in the copolymers [34].

#### 3.2.7. Antimicrobial Activity

**Figure 6:**
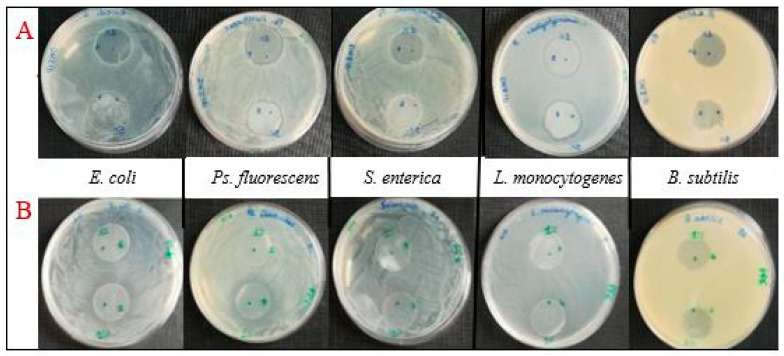
Images of inhibition halos induced by Pebax^®^Rnew/IL1 (**A**) and Pebax^®^Rnew/IL2 (**B**) blends, after 24 h of incubation on NA plates inoculated with 100 µL cell suspension at 10^6^ CFU/mL concentration of *E. coli*, *Ps. fluorescens*, *S. enterica*, *L. monocytogenes* and *B. subtilis*.

The ILs were tested for their antimicrobial activity against Gram-negative (*E. coli*, *Ps. fluorescens*, *S. enterica*) and Gram-positive (*L. monocytogenes* and *B. subtilis*) bacteria. Table 4 reports the width (mm) of the inhibition zones (average of three independent replicates ± standard deviation) induced by Pebax^®^Rnew/ILs films (1 and 5 wt.%) on NA plates inoculated with the above mentioned strains. Figure 6 displays images of the obtained inhibition halos.

Pebax^®^Rnew/IL1 films displayed a good antimicrobial activity against *Ps. fluorescens* and *L. monocytogenes* growth at both loading concentrations (1 and 5%), whereas only the blend containing 5 wt.% of IL1 showed a weak inhibitory activity against *E. coli* growth. These results suggest that antibacterial activity against *E. coli* of IL1 is dependent on its content into polymer matrix although, against the other pathogens, good results were obtained even at low concentrations. 

Pebax^®^Rnew/IL2 exerted antibacterial activity towards *E. coli* at both concentrations, showing wider halos at increasing concentrations into polymer matrix. At 1% wt. concentration, IL2 showed a lack of inhibition toward *Ps. fluorescens* that was clearly inhibited only when IL2 was tested at the highest content (5 wt.%). Both Pebax^®^Rnew/IL1 and Pebax^®^Rnew/IL2 films did not exhibit antimicrobial activity against *S. enterica* and *B. subtilis* strains within the tested incubation time.

Control plates made with neat Pebax^®^Rnew did not show any inhibition of bacterial growth. 

Despite the films did not exert antibacterial activity against *S. enterica* and *B. subtilis*, it is not excluded that Pebax^®^Rnew/IL1s films may show antimicrobial properties when the pathogen is present at lower concentrations or in matrices different from the culture medium used. Our results proved that bacterial sensitivity increases for longer IL chain lengths, being high in the case of IL1, in agreement with the reported antimicrobial activity of ILs having longer alkyl chains in the imidazolium cation [35,36]. The antimicrobial activity of blend films containing IL2 at 5% concentration is higher than those loaded with IL1, probably due to its massive release, as shown below. Nevertheless, antimicrobial activity of these blends decreased from 5 to 1% concentrations, showing a clear dependence on concentration. This behavior was not observed for IL1.

IL1, [Hdmim^+^] [DMSIP^−^], was previously used for the synthesis of imidazolium poly(butylene terephthalate) ionomers with ionic groups located selectively as covalent and ionic end-groups (telechelic) or randomly along the polymer chain [10]. In agreement with our results, these ionomers displayed antimicrobial activity generally higher (about double) against *S. aureus* (Gram+ bacteria) than *E. coli* (Gram− bacteria). However, we also found antimicrobial activities vs. both L. monocytogenes (Gram+) and Ps. fluorescens (Gram−) very similar, confirming that antimicrobial activity mainly depends on bacterial strain sensitivity (and not only on cell wall structure).

With regard to the IL2, it has not been used previously in polymer blends. Indeed, Pernak et al. [21] analyzed the minimum inhibitory concentration (MIC) values and minimum bactericidal or fungicidal concentration (MBC) of a series of pure ILs bearing alkoxymethyl chains. They found that antimicrobial activity of ILs are greatly affected by the alkyl chain length in the alkoxymethyl substituent, as the short substituents (propoxymethyl, butoxymethyl and pentyloxymethyl) are not active against bacteria and fungi.

#### 3.2.8. IL Release

**Figure 7:**
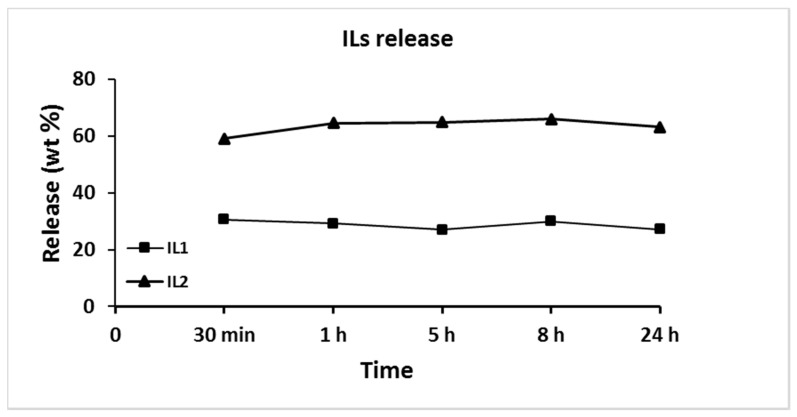
ILs release in water from Pebax^®^Rnew/ILs blends containing ILs at a concentration of 5 wt.%.

The ILs release (weight percentage) was determined after 30 min, 1, 5, 8 and 24 h of immersion of blends films loaded with 5% of ILs in sterile water. The percentages of ILs migration after the fixed periods of incubation are shown in Figure 7.

The films display a release of the antibacterial agents already at short timescales of action. In particular, the ILs release occurs during the first 30 min (burst behavior in the initial stage) and is stable during the 24 h. However, the two ILs showed different released amounts in the 24 h: 30% for Pebax^®^Rnew/IL1 and 67% for Pebax^®^Rnew/IL2 blends, respectively. These large amounts suggest low or no miscibility between the polymer and the ILs, with consequent accumulation of ILs on polymer surface. These results are in agreement with those from SEM analyses, confirming the phase separation discussed in the DSC data analysis, particularly in the case of IL2 with the Rnew polymer matrix. The ILs easily and quickly migrate from surface blends due to weaker interaction with the polymer, principally for the smaller IL2. In our study, the films loaded with IL2, having a larger gas permeability, allowed a more intense exchange with the environment, and thus a greater release in the water medium. Therefore, the polymeric matrix fails to retain the IL2 within its polymeric network. Release is lower for the bulkier IL1 (longer alkyl tail in the cation and bigger anion), owing to a lower diffusion rate within the polymeric matrix.

This is consistent with other studies on polymeric films incorporating imidazolium-based ILs [29]. Rogalsky and co-authors prepared polymeric films of PC loaded with ILs having different alkyl chain lengths in the cation and the same anion. The films showed high activity against the *E. coli* strain, with a clear dependence on the IL content and without effect of alkyl chain length of ILs imidazolium cation [29]. They supposed that the more active IL comprising longer alkyl chains had a lower release rate from the PC film than ILs with shorter alkyl moieties.

#### 3.2.9. Cytotoxicity of Pebax^®^Rnew/ILs Blends

**Figure 8:**
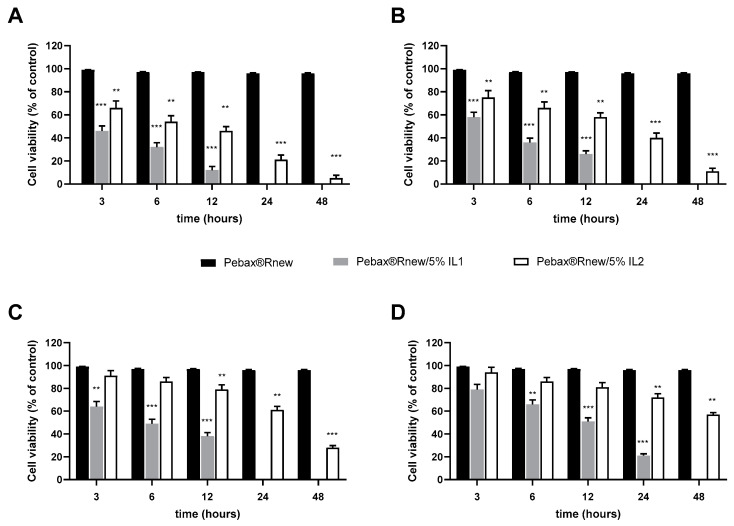
Effect of ILs on cell viability. Caco-2 cells were treated with ILs released medium serially diluted by a factor of 10: (**A**) 1, (**B**) 0.1, (**C**) 0.01 and (**D**) 0.001. Cell viability was assessed by CCK-8 assay after 3, 6, 12, 24 and 48 h of incubation at 37 °C. ILs cytocompatibility was expressed as a percentage relative to untreated cells used as control. Data are presented as the mean ± SD for six independent measurements (*n* = 6). ** *p* < 0.01, *** *p*< 0.001 respect to Pebax^®^/Rnew.

Although ILs are considered green chemicals able to replace volatile organic solvents currently used by industry, few studies have reported the toxicological effects of these materials in in vitro mammalian cell line models. The cytotoxicity occurs via various mechanisms leading to cell death via necrosis or apoptosis [37,38]. Because the first contact of an organism with toxic compounds generally takes place with the epithelial cells, in this study, Caco-2 cell lines were used as in vitro model to evaluate the cytotoxicity of ILs released from Pebax^®^Rnew blends. As showed in Figure 8, the inhibitory effect on the cell viability is time- and dose-dependent. IL1 presents a stronger adverse effect, even at the highest dilution at the all-times (Figure 8D), probably due to the long alkyl chain. On the contrary, IL2 showed a lower cell toxicity, even at low released medium dilution according to the literature data. In fact, ILs toxicity decreases with the decrease of the alkyl chain length and the incorporation of an oxygen atom in the side chains of the imidazolium cation [39]. These data are also consistent with those reported for ILs, analyzing the 1-octanol/water partition coefficient that describes the hydrophobic character of a substance and is recognized as a toxicological parameter to assess some ecosystem risk factors such as bioaccumulation. It increases with the alkyl chain length in imidazolium ILs, evidencing a higher hydrophobicity [40], whereas with the introduction of polar functionalized ether or hydroxyl groups into alkyl chain it decreases. Typically, larger values of the 1-octanol/water partition coefficient are correlated with bioaccumulation and toxicity in fish and/or to adsorption in soils [41].

Considering these results, further studies on the use of lower IL1 concentrations, whose antimicrobial activity did not depend on dose, could be useful to improve knowledge on its biocompatibility and cytotoxicity.

The developed Pebax^®^/Rnew IL1 blend membranes reversed the wettability of the polymer, providing a better antimicrobial performance with a good combination of hydrophilicity, permeability and thermal stability. Attractive aspects of these novel antimicrobial films are represented by the use of a biocompatible thermoplastic poly(ether-b-amide) copolymer that comprises a bio-based polyamide and is highly permeable and flexible and of a green solvent [42]. Indeed, the most studied biodegradable materials such as poly(lactic acid) or poly(glycolic acid) produce acidic degradation products that may accumulate, leading to toxicity [43]. The use of a rubber copolymer, avoiding hazardous plasticizers, is highly suited for many application fields. Results on ILs release and cytotoxicity by Pebax^®^Rnew/ILs blend films, however, suggest to avoid their application in contact with water at high ILs concentrations (5%). In light of this, possible application fields of the manufactured films could be surface antimicrobial coating on furniture for sterile environments/rooms and filters for aerating systems. However, at low concentrations of ILs (≤1%), their use could also be extended to other sectors, such as the biomedical field.

## 4. Conclusions

Imidazolium-based ILs were synthetized and incorporated (at 1 and 5 wt.%) into the elastomeric polyether block PA11 (Pebax^®^Rew). Flexible films were obtained by solution casting technique owing to the good film-forming properties of the chosen polymeric matrix, without the necessity of plasticizers. Thermal analysis indicates a good thermal resistance for the blends and phase separation between the copolymer and the ILs. Gas permeation rate tests show the poreless nature of the prepared films with moderate permeability, slightly reduced upon the incorporation of the ILs that are solid at room conditions.

The ILs loaded films, even with small amount of ILs, can be developed for inhibiting the growth of prominent pathogenic bacteria such as *E. coli* and *L. monocytogenes*. They provide a good short-term antimicrobial effect by releasing most of the incorporated ILs in the first 24 h. The mechanism of IL release from the films depends on the salt molar volume, being more significant for the smaller IL2 salt ([OOMmim][PF_6_]). The antimicrobial activity depends on the substitution pattern of the imidazolium cation and on the anion type. IL2-loaded films have no antimicrobial toxicity against Gram-positive bacteria, showing a high uncontrolled release in water. On the other hand, IL1 ([Hdmim][DMSIP]) blends display better antimicrobial activity on tested Gram-positive and Gram-negative bacteria owing to the longer alkyl chain on the cation, a moderate release in water with a burst initial stage at short times. Moreover, IL1 is capable to trigger the surface hydrophilicity of the samples, leading to attractive antimicrobial films. Nevertheless, data on ILs released from Pebax^®^Rnew/ILs blends and on their biocompatibility/cytotoxicity suggest their application as antimicrobial coating on furniture for sterile environments/rooms and as filters for aerating systems.

## Figures and Tables

**Table 1:** Melting temperatures (*T*m) and enthalpy of fusion (∆*H*m) of PTMO and PA blocks in the second heating run of Pebax^®^Rnew and Rnew/ILs blends.

Sample	PTMO		PA11	
	*T*m (°C)	Δ*H*m (J g^−1^)	*T*m (°C)	Δ*H*m (J g^−1^)
**Neat Pebax^®^Rnew 25R53**	12	27.8	142	4.0
**Pebax^®^Rnew—1% IL1**	14	15.9	137	4.2
**Pebax^®^Rnew—5% IL1**	(−25) 14	(6.4) 4.1	137	3.5
**Pebax^®^Rnew—1% IL2**	13	14.3	134	3.6
**Pebax^®^Rnew—5% IL2**	17	17.2	129	3.4

**Table 2:** Thermogravimetric data of neat Pebax^®^Rnew and its blends.

Samples	T_Δ__m= 5%_ (°C) ^a^	T_d1_ (°C) ^b^	% R ^c^
**Neat Pebax^®^Rnew 25R53**	366	418	0.0
**Pebax^®^Rnew—1% IL1**	337	413	0.2
**Pebax^®^Rnew—5% IL1**	333	402	0.1
**Pebax^®^Rnew—1% IL2**	354	422	0.2
**Pebax^®^Rnew—5% IL2**	222	415	0.0

^a^: Onset of degradation (temperature of 5% weight loss); ^b^: Decomposition maximum temperature of thermal degradation; ^c^: Weight residue (%) at 600 °C.

**Table 3:** Gas permeability of Pebax^®^Rnew and Pebax^®^Rnew/IL blend membranes at 25 °C.

IL Type (wt.%)_	Permeability (Barrer)				Selectivity (–)	
Thickness	CO_2_	O_2_	N_2_	He	CO_2_/N_2_	O_2_/N_2_
Neat Polymer _120 micron	247	23.6	8.9	25.7	27.9	2.66
**IL1 (1%)** 115 micron	217	21.4	8.5	23.7	25.6	2.53
**IL1 (5%)_** 80 micron	195	19.2	7.2	24.1	27.2	2.68
**IL2 (1%)** 90 micron	232	22.4	8.6	25.7	27.0	2.60
**IL2 (5%)** 100 micron	226	21.6	8.1	25.8	28.0	2.61

1 Barrer = 10^−10^ cm^3^ (STP, standard temperature and pressure) cm cm^−2^ cmHg^−1^ s^−1^.

**Table 4:** Inhibition halos (mm) induced by Pebax^®^Rnew/ILs films, after 48 h of incubation, on nutrient agar (NA) plates containing bacterial strains.

Bacteria Strain		Inhibition Halos (mm) Induced by Pebax^®^Rnew/ILs Films			
		Pebax^®^Rnew/IL1		Pebax^®^Rnew/IL2	
		1 wt.%	5 wt.%	1 wt.%	5 wt.%
Gram−	*E. coli*	-	1 ± 0.0	1 ± 0.0	4 ± 0.5
	*Ps. fluorescens*	3 ± 0.2	3 ± 0.5	-	5 ± 0.5
	*S. enterica*	-	-	-	-
Gram+	*L. monocytogenes*	3 ± 0.5	3 ± 0.2	-	-
	*B. subtilis*	-	-	-	-

## Supplementary Materials

The following are available online at https://www.mdpi.com/2073-4360/12/6/1419/s1, Figure S1: 1HNMR spectrum of [Hdmim+] [DMSIP-] (IL1); Figure S2: 1HNMR spectrum of [OOMmim+] [PF6-] (IL2); Figure S3: MALDI-TOF Mass spectrum of [Hdmim+] [DMSIP-] (IL1); Figure S4: MALDI-TOF Mass spectrum of [OOMmim+] [PF6-] (IL2); Figure S5: DSC curves of [Hdmim+] [DMSIP-] (IL1); Figure S6: DSC curves of [OOMmim+] [PF6-] (IL2); Figure S7: FTIR spectra for neat Pebax^®^Rnew and blends at 5 wt.% loading of ILs.
