# Supplementary material for: Heterogenized Imidazolium-Based Ionic Liquids in Pebax®Rnew. Thermal, Gas Transport and Antimicrobial Properties
Source: Polymers (Basel). 2020 Jun 25;12(6):1419. doi: 10.3390/polym12061419 (PMC7361949; doi:10.3390/polym12061419)
Supplement: Supplementary file 1 [file polymers-12-01419-s001.pdf]

Supporting information

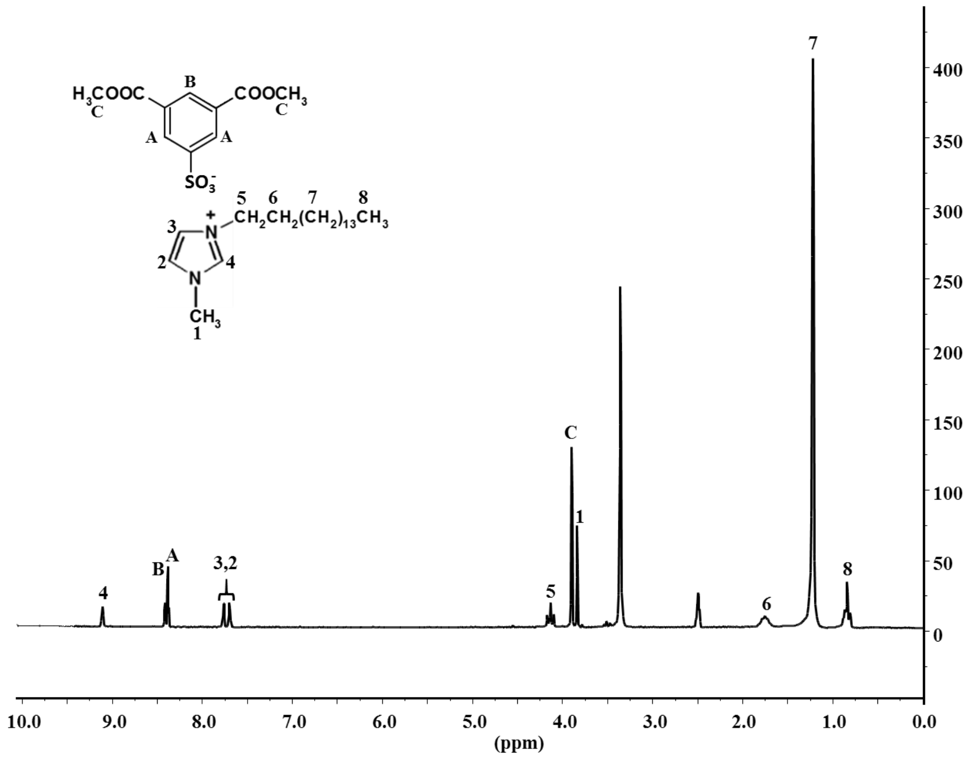

Fig. 1 SI. ^{1}H NMR spectrum of Hdmim+ DMSIP- (IL1).

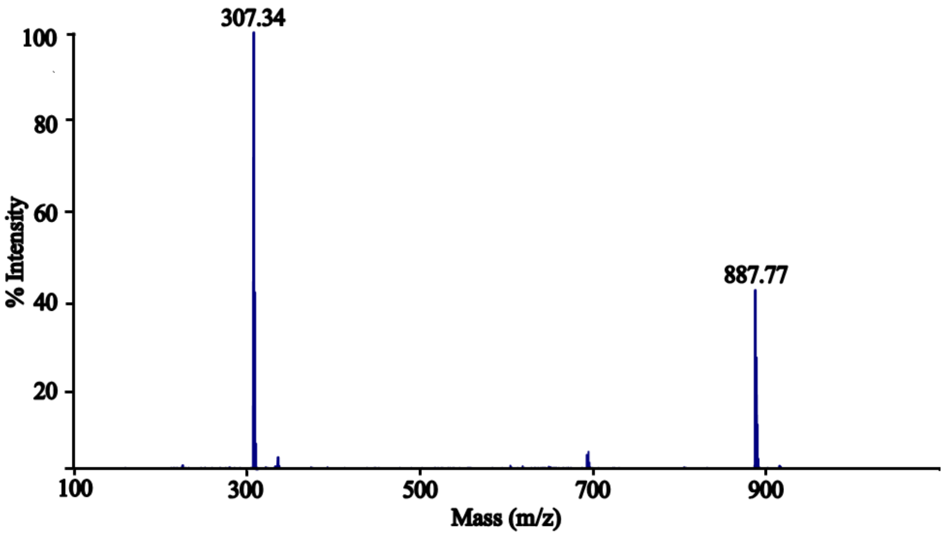

Fig. 2 SI. MALDI spectrum of $Hdmim^{+}$ $DMSIP^{-}$ (IL1).

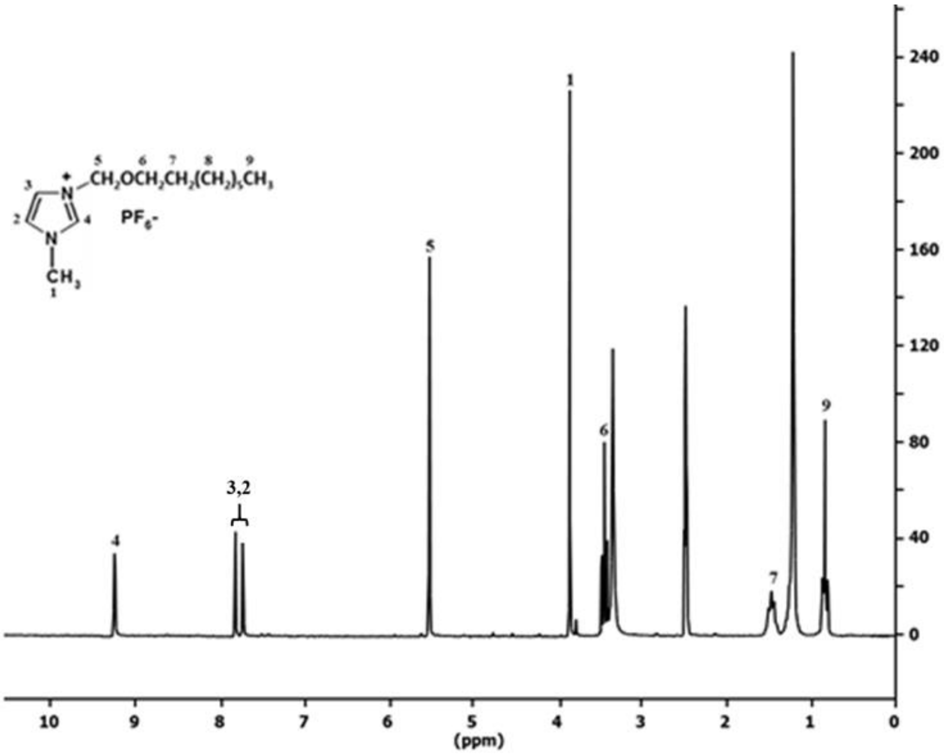

Fig. 3 SI. ^{1}H NMR OOMmim$^+$ PF$_6^-$ (IL2).

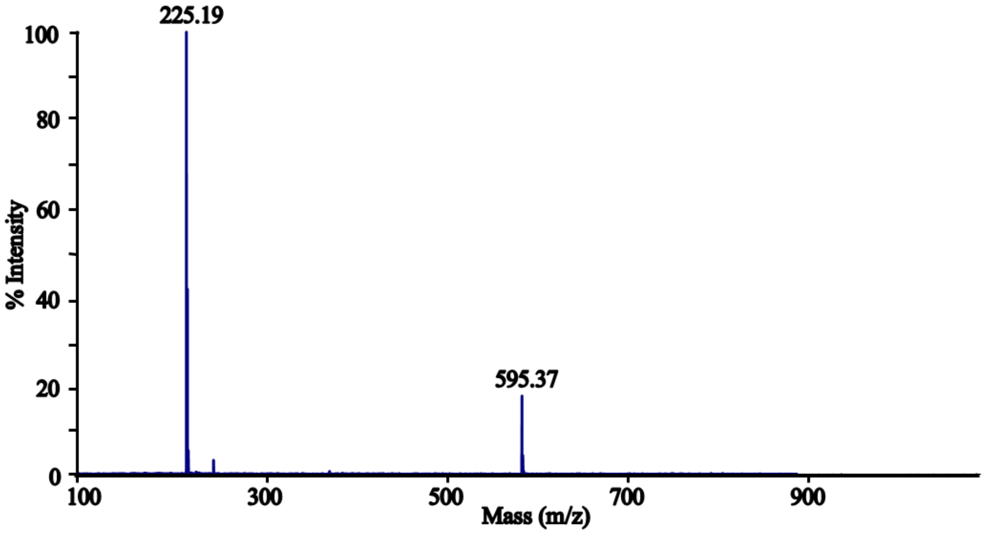

Fig. 4 SI. MALDI spectrum of OOMmim$^+$ PF$_6^-$ (IL2).

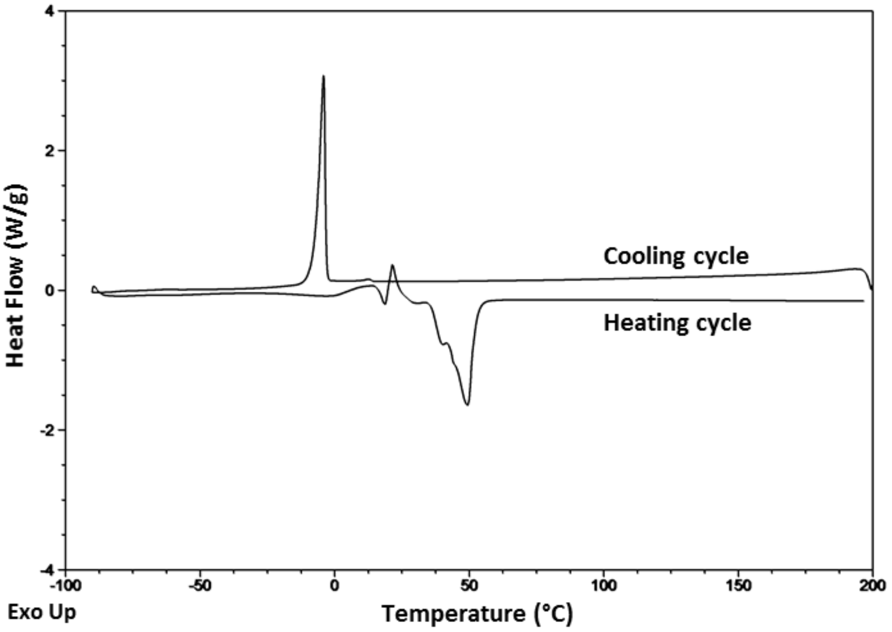

Fig. 5 SI. DSC of $Hdmim^{+}$ $DMSIP^{-}$ (IL1).

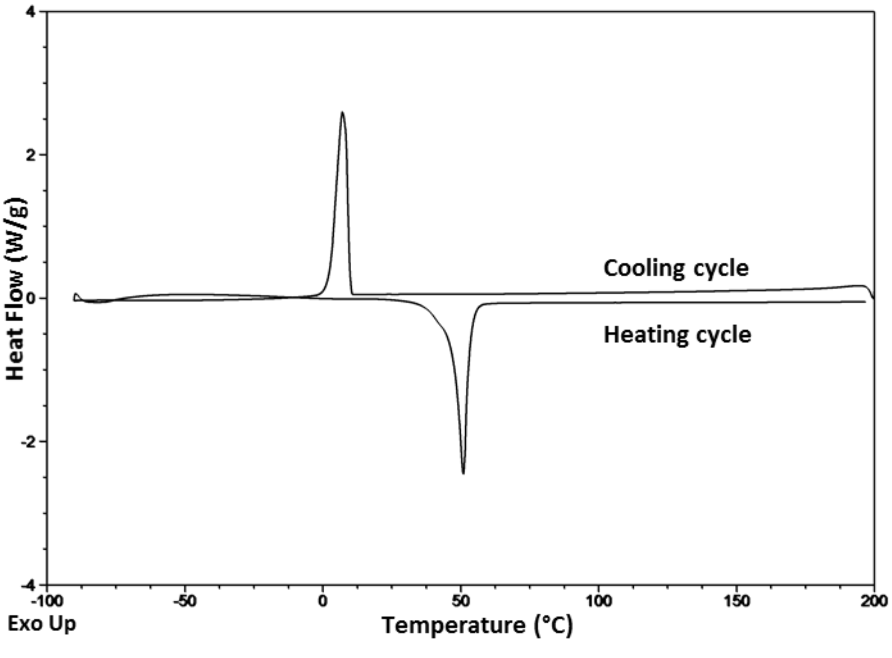

Fig. 6 SI. DSC $OOMmim^{+}$ PF_6^{-} (IL2).

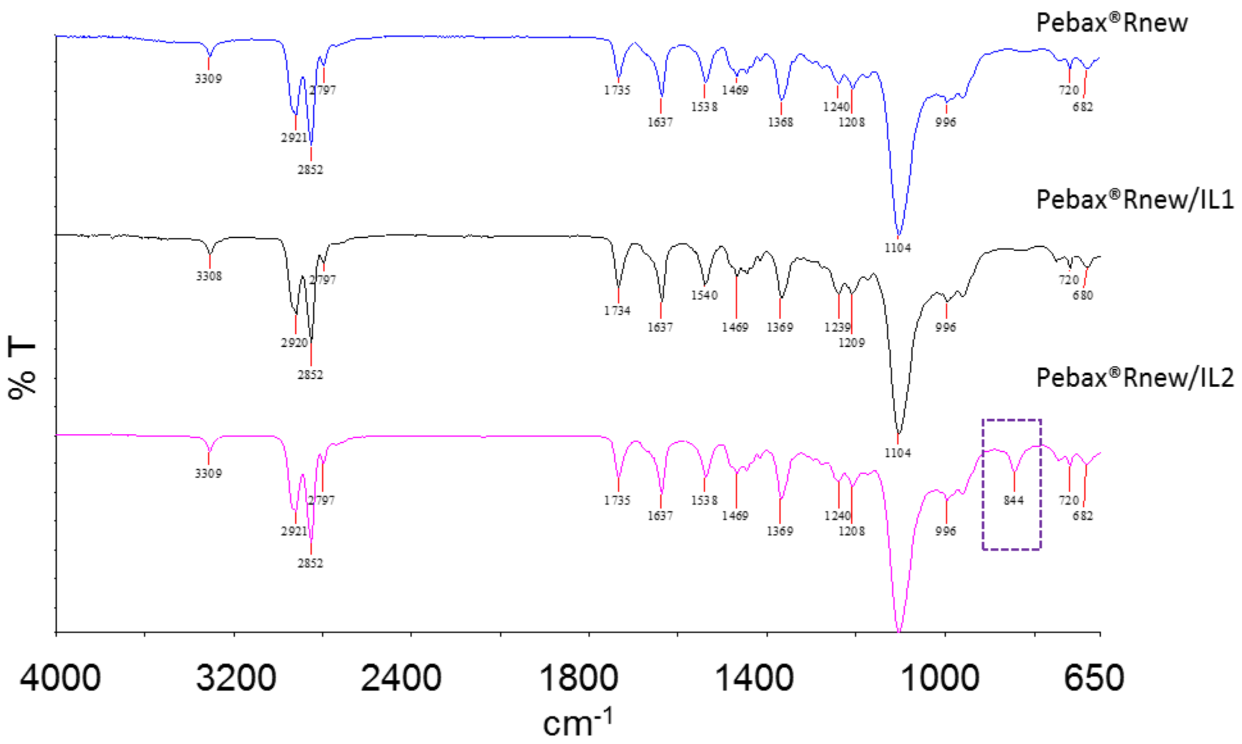

Fig. 7 SI. FTIR spectra for neat Pebax®Rnew and blends at 5 wt.% loading of ILs.
